# Baculovirus-mediated gene transfer and recombinant protein expression do not interfere with insulin dependent phosphorylation of PKB/Akt in human SHSY-5Y and C3A cells

**DOI:** 10.1186/1471-2121-8-6

**Published:** 2007-02-19

**Authors:** Monica Andersson, Malin Warolén, Joakim Nilsson, Martin Selander, Catharina Sterky, Katrin Bergdahl, Christina Sörving, Stephen R James, Magnus Doverskog

**Affiliations:** 1 Department of Biology, R&D, Biovitrum AB, SE-11276 Stockholm, Sweden; 2 Department of Lead Discovery, R&D, Biovitrum AB, SE-11276 Stockholm, Sweden; 3 Preclinical Development, R&D, Biovitrum AB, SE-11276 Stockholm, Sweden; 4 Discovery Research, R&D, Biovitrum AB, SE-11276 Stockholm, Sweden; 5 Process Development, Biopharmaceuticals, Biovitrum AB, SE-11276 Stockholm, Sweden

## Abstract

**Background:**

Recombinant adenovirus vectors and transfection agents comprising cationic lipids are widely used as gene delivery vehicles for functional expression in cultured cells. Consequently, these tools are utilized to investigate the effects of functional over-expression of proteins on insulin mediated events. However, we have previously reported that cationic lipid reagents cause a state of insulin unresponsiveness in cell cultures. In addition, we have found that cultured cells often do not respond to insulin stimulation following adenovirus treatment. Infection with adenovirus compromises vital functions of the host cell leading to the activation of protein kinases central to insulin signalling, such as protein kinase B/Akt. Therefore, we investigated the effect of adenovirus infection on insulin unresponsiveness by means of Akt activation in cultured cells. Moreover, we investigated the use of baculovirus as a heterologous viral gene delivery vehicle to circumvent these phenomena. Since the finding that baculovirus can efficiently transduce mammalian cells, the applications of this viral system in gene delivery has greatly expanded and one advantage is the virtual absence of cytotoxicity in mammalian cells.

**Results:**

We show that infection of human neuroblastoma SHSY-5Y and liver C3A cells with recombinant adenovirus results in the activation of Akt in a dose dependent manner. In addition, this activation makes treated cells unresponsive to insulin stimulation as determined by an apparent lack of differential phosphorylation of Akt on serine-473. Our data further indicate that the use of recombinant baculovirus does not increase the phosphorylation of Akt in SHSY-5Y and C3A cells. Moreover, following infection with baculovirus, SHSY-5Y and C3A cells respond to insulin by means of phosphorylation of Akt on serine-473 in the same manner as uninfected cells.

**Conclusion:**

Widely-used adenovirus vectors for gene delivery cause a state of insulin unresponsiveness in human SHSY-5Y and C3A cells in culture due to the activation of central protein kinases of the insulin signalling pathway. This phenomenon can be avoided when studying insulin signalling by using recombinant baculovirus as a heterologous viral expression system. In addition, our data may contribute to an understanding of the molecular mechanisms underlying baculovirus infection of human cells.

## Background

The insulin receptor (IR) is a heterotetrameric protein tyrosine kinase whose kinase activity is activated upon binding of insulin [1]. Several aspects of cell metabolism and physiology are controlled and regulated by insulin, and through interactions with the IR at the cell surface, insulin causes the modulation of several interacting signal transduction pathways subsequently leading to physiological responses at the cellular level. The function of the transduction pathways is to coordinate the activation or inactivation of proteins in response to external stimuli. Moreover, pleiotropic [2] and differential properties, both in terms of common steps in their pathways [3] as well as in differential activation [4] make signalling diverse. Protein phosphorylation and dephosphorylation play a major role in intracellular signal transduction regulation [5] and the phosphorylation state of a target protein is dependent on the equilibrium between these activities. Disturbances in any element of such regulatory systems may lead to pathological conditions such as diabetes type II and cancer and are therefore the subject of extensive studies. In our research work we have performed various studies which investigate the effects of over-expression of a variety of different proteins on insulin-mediated events in cells in culture. The outcome of such studies places an emphasis on the ability to perform quantitative analyses with low back-ground noise.

Gene delivery into mammalian cells for protein over-expression and assessment of gene function involves viral as well as non-viral methods. Commonly, the non-viral methods comprise plasmid transfection with cationic lipids or polymers [6]. In contrast, viral transduction utilises gene transfer by infection of host cells with a modified virus (reviewed in [7]). Advantages of using viral delivery systems are related to less cell membrane damage and a higher degree of transduction efficiency as compared to the non-viral methods, thus providing viruses as excellent tools for gene delivery to study cell biological processes.

Nevertheless, the use of viral delivery systems may suffer from lengthy construction time requiring transfection steps, cloning of producer cell lines to generate virus stocks, stock amplification and purification. In addition, homologous viral systems of animal or human origin, such as the commonly utilized adenovirus expression system, must be engineered to remove functions involved in the expression of viral genes and viral replication [8]. In addition, the use of recombinant viruses for gene delivery comprises vital functions such as cell membrane adhesion and entry, cytoplasmic transport, replication of viral genome (DNA viruses), and subsequently transcription and translation of the gene of interest (adenovirus endocytosis reviewed in [9]). It is well documented that such mechanisms involved in adenovirus infection and host inflammatory response modulate several host cell signalling pathways [10] which therefore may interfere with functional mechanisms in study. For example, incoming adenoviruses have been shown to up-regulate two distinct cell signalling pathways leading to the activation of integrins and cAMP-dependent proteinkinase A (PKA), and p38/MAP kinase pathways, respectively [11]. Downstream of p38/MAP kinase, MAPKAP kinase 2 (MK2) was shown to be activated upon adenovirus infection [11]. Interaction of adenovirus with α_v _integrins induces the activation of phosphatidylinositol 3-kinase (PI3-kinase) which triggers endocytosis [12,13]. Downstream of PI3-kinase, both protein kinase B/Akt and ERK/MAP kinase signalling pathways have been shown to be activated in a dose-dependent manner within minutes following adenovirus infection [14,15]. In addition, the phosphorylation of glycogen synthase kinase (GSK)-3β and nuclear translocation of the p65 subunit of NF-κB, both downstream targets of the PI3-kinase/Akt pathway, was demonstrated in adenovirus-infected corneal fibroblasts [16]. Furthermore, it was shown that adenovirus overrides cellular protein translation in a process involving the downstream activation of mTOR [17]. Similarly, downstream of the p38/MAP kinase pathway, MAP kinase interacting kinase 1 (Mnk1) [18,19] was displaced from the eukaryotic initiation factor 4F (eIF4F) complex in HEK293 cells, thereby blocking the phosphorylation of eIF4E by Mnk1 and the subsequent translation of cellular mRNA in HEK293 cells [20].

Over the past 20 years, the insect cell/baculovirus expression vector system, BEVS [21], has become an important tool for laboratory as well as for industrial scale expression of various heterologous proteins [22-24]. BEVS has also emerged as a versatile and powerful expression system for functional studies of proteins such as protein kinases [25] and G protein-coupled receptors [26], for protein-protein interactions [27], cell based screening [28] and for viral surface display [29]. Moreover, BEVS has been shown to serve as an efficient gene-transfer vehicle in mammalian cells (termed BacMam) for transient and stable expression of recombinant proteins [30]. Lately, there has been an increasing interest in the use of baculovirus for gene delivery in mammalian cells for protein production, functional expression, and gene therapy (reviewed in [31-33]. For example, the BacMam expression system was recently used to study estrogen receptor function in osteosarcoma cells [34], functional characterization of a K_ATP _channel protein in CHO and HEK293 cells [35], and for RNA interference in human primary cells [36]. It has also been developed for the application of nuclear- [37,38] and G protein-coupled-receptor [39, 40] drug discovery. This interest has been enthused by the relative ease of construction and propagation of recombinant baculovirus vectors, restricted host range for viral replication and viral gene expression, combined with high transduction efficiency and no to low observable cytotoxicity in a wide range of mammalian cell types (reviewed in [41]). The use of specially engineered recombinant baculoviruses as efficient vehicles for gene transfer into mammalian cells is rapidly emerging as a powerful system for a variety of applications.

In this work we have investigated the use of engineered *Autographa californica *multiple nuclear polyhedrosis virus (AcMNPV) as a gene delivery tool to study insulin mediated events in human neuroblastoma SHSY-5Y and hepatic C3A cells. Previously, investigating the effects of over-expression of proteins on insulin mediated events, we found that cells in culture often became resistant to stimulation with insulin subsequent to treatment with transfection agents comprised of cationic lipid preparations [42]. This was shown to be caused by an induced state of insulin unresponsiveness due to insulin receptor activation and subsequent down-regulation. Similarly, we have found that cells in culture often become resistant to stimulation with insulin subsequent to treatment with adenovirus vectors (unpublished). In the present work we highlight the fact that adenovirus infection of human cells in culture involves the activation of PI3-kinase and subsequently the downstream activation of Akt. The PI3-kinase/Akt pathway is central to insulin mediated signalling and Akt is central for cellular responses mediated by insulin (reviewed in [43]). We show that adenovirus mediated gene transfer in human neuroblastoma SHSY-5Y and liver C3A cells cause a dose dependent phosphorylation of Akt and that this makes the cells unresponsive to insulin. Furthermore, we suggest that this phenomenon can be avoided by using recombinant AcMNPV baculovirus mediated gene transfer when studying insulin signalling in human neuroblastoma or liver cells.

## Results

### Infection of human neuroblastoma SHSY-5Y and liver C3A cells with recombinant adenovirus induces Akt-Ser473 phosphorylation

**Figure 1:**
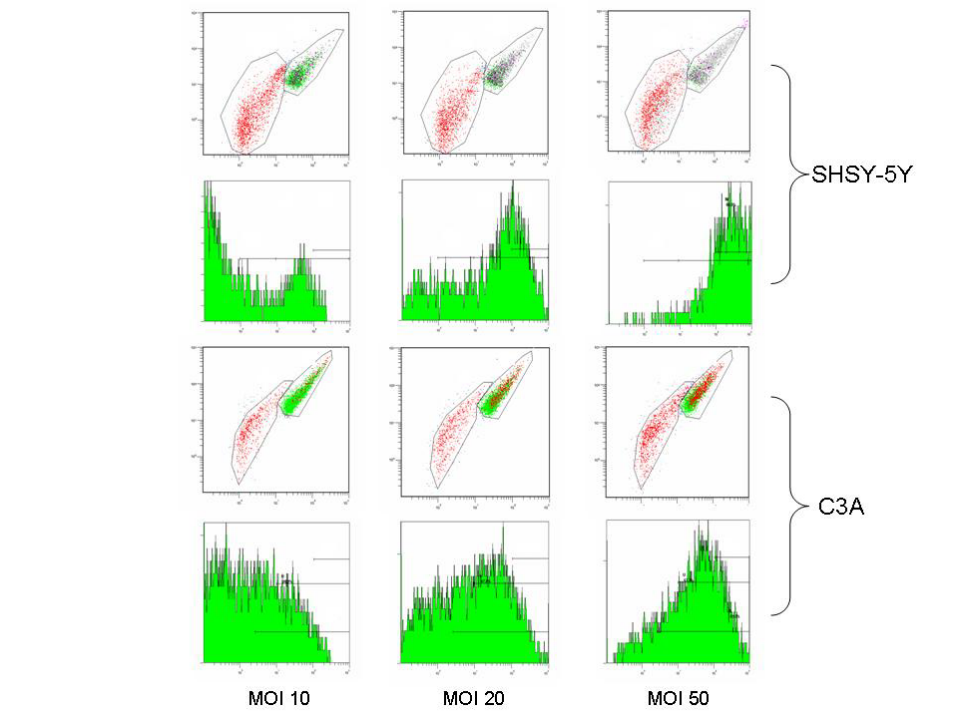
**Transduction of SHSY-5Y and C3A cells 48 hours post infection with recombinant adenovirus expressing green fluorescent protein**. Flow cytometry scatter plots of SHSY-5Y and C3A cells infected at MOI 10, 20, and 50; cells corresponding to the upper areas of the respective scatterplot (upper panels) were analysed for transduction efficiency by GFP expression (lower panels).

Adenoviral gene delivery is commonly utilized for functional over-expression and characterization of a gene-of-interest in mammalian cells. However, it is widely recognized that viral infection of human cell lines with homologous adenovirus vectors induces the activation of phosphatidylinositol 3-kinase (PI3-kinase), subsequently leading to the phosphorylation of protein kinase B/Akt on Serine 473 [12,11,14]. Akt is central for cellular responses mediated by insulin (reviewed in [43]) and activation of Akt is hence used as a common marker in insulin signalling. Therefore, while studying the impact of over-expression of various protein kinases on insulin signalling in experimental human cell lines, we investigated the influence of recombinant adenovirus on Akt phosphorylation in SHSY-5Y and C3A cells. Cultured cells were first infected with recombinant adenovirus expressing green fluorescent protein (AdGFP) as a model protein, and phosphorylation of Akt was studied with specific Ser^473^-phosho-Akt antibodies (ELISA and western blot). Viral titration was performed at multiplicity of infection (MOI) between 10 and 50 and GFP expression was determined 48 hours post infection (p.i.) by flow cytometry gated for intact adenovirus infected cells. Figure 1 displays side scatter against forward scatter for SHSY-5Y and C3A cells infected with AdGFP at MOI 10, 20, and 50, respectively. The resulting transduction efficiencies were determined from expression histograms of the populations of intact cells (illustrated as the upper cell population in respective scatter plot) which are shown in the lower panels of Figure 1 for each respective cell line, and was found to be 26.5, 58.6, and 86.9 % for SHSY-5Y cells, and 26.2, 47.8, and 67.3 % for C3A cells infected at MOI 10, 20, and 50, respectively.

**Figure 2:**
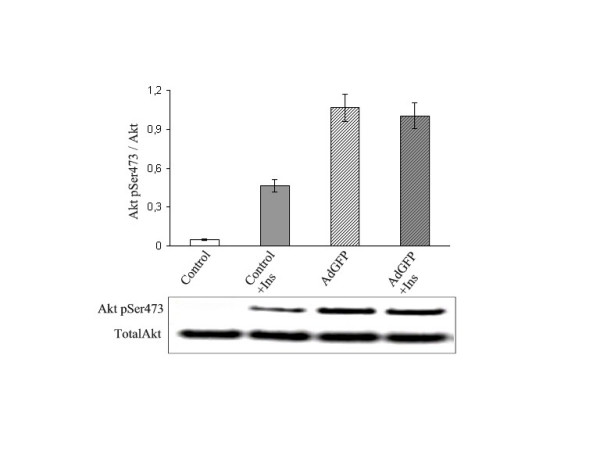
**Adenovirus vectors activate Akt kinase and make cells unresponsive to insulin**. Cultured SHSY-5Y cells were treated with 100 nM insulin alone for 5 minutes (Control + Ins), or infected with recombinant adenovirus alone at MOI20 (AdGFP), respectively, and compared to cells similarly treated with insulin but pre-infected with adenovirus 48 h previously (AdGFP + Ins). Cells were harvested for transduction efficiency and total cellular protein, and Western blot analyses were performed with antibodies specific for Akt phosphorylated on serine 473 and total Akt, respectively (lower panel). For quantification, densitometry of the Western blot data were performed (method S.D. < 10%, n = 3).

Next, the influence on Akt phosphorylation following adenovirus transduction was investigated. As anticipated, the degree of phosphorylation of Akt increased upon adenovirus infection and this increase was dose-dependent when both SHSY-5Y as well as C3A cells were subjected to adenovirus infection. The degree of phosphorylation increased 4 and 5-fold for SHSY-5Y cells, and 1.8 and 2.4-fold for C3A cells, infected at MOI 10 and 50, respectively, as determined with an ELISA assay measuring phosphoserine 473 in PKB. These results were confirmed under various experimental conditions and are summarized in the upper part of Table 1 for AdGFP. To further validate the results, the data obtained with the commercial ELISA were verified with western blot using specific Ser^473^-phosho-Akt antibodies. Again, phosphorylation on serine 473 in infected but unstimulated cells was seen, thus confirming the activation of PKB by adenovirus infection measured by two different methods (Figure 2, lane 1 and 3). Taken together, the results from these experiments show that when transduced with adenovirus up to MOI 50, SHSY-5Y and C3A cells respond with an increased phosphorylation of Ser473 Akt indicating that using adenovirus as a gene transfer vehicle in SHSY-5Y and C3A cells may influence the cells responsiveness to insulin treatment.

**Table 1:** Influence of transduction with recombinant adenovirus (AdGFP) or baculovirus (BacGFP) on phosphorylation of Akt Ser-473 in SHSY-5Y and C3A cells.

**Cells**	**Virus**	**MOI**	**Akt **(A.U.)		**pSer473 Akt **(A.U.)		**pSer473 Akt/Akt**	
			10 min	30 min	10 min	30 min	10 min	30 min
			
SHSY-5Y	AdGFP	0	2.73	2.46	0.20	0.32	**0.07**	**0.13**
SHSY-5Y	AdGFP	10	2.60	2.24	0.81	0.90	**0.31**	**0.40**
SHSY-5Y	AdGFP	50	2.25	2.03	0.80	1.30	**0.35**	**0.64**
								
C3A	AdGFP	0	0.80	0.95	0.15	0.24	**0.19**	**0.25**
C3A	AdGFP	10	0.72	0.64	0.25	0.45	**0.35**	**0.70**
C3A	AdGFP	50	0.69	0.59	0.31	0.54	**0.45**	**0.91**

SHSY-5Y	BacGFP	0	2.64	2.56	0.25	0.36	**0.09**	**0.14**
SHSY-5Y	BacGFP	1000	2.74	2.62	0.20	0.40	**0.07**	**0.15**
SHSY-5Y	BacGFP	10000	2.86	2.69	0.22	0.36	**0.08**	**0.13**
								
C3A	BacGFP	0	0.94	0.79	0.16	0.20	**0.17**	**0.25**
C3A	BacGFP	1000	1.04	0.84	0.15	0.20	**0.15**	**0.23**
C3A	BacGFP	10000	1.09	0.89	0.15	0.23	**0.14**	**0.26**

Time notations refer to time of incubation at experimental conditions determined by the ELISA method.

### Baculovirus mediated gene transduction and recombinant protein expression do not influence the level of phosphorylation of Akt in human SHSY-5Y and C3A cells

The use of engineered baculovirus as a gene transfer vehicle in mammalian cells has emerged as a powerful tool for several applications. One advantage of using this heterologous viral system is the virtual absence of endogenous viral gene expression and cytotoxicity in mammalian cells [32]. In view of the results obtained above, we therefore investigated the use of baculovirus as a potential tool for gene delivery in human cell lines used to study insulin signalling. High efficiency baculovirus transduction of various liver [44,45] and neural cell lines [46] has been established previously. However, the use of the human neuroblastoma SHSY-5Y cells has to our knowledge not been reported and transduction efficiency and expression of reporter protein in SHSY-5Y cells was therefore first confirmed. Similar to that for adenovirus (AdGFP), a viral construct containing GFP under the control of a cytomegalovirus promoter (CMV) was first constructed (BacGFP). Recombinant virus was propagated in Sf9 insect cells and prepared as described in the Methods section. Adherent cultures of SHSY-5Y cells were infected with BacGFP and expression of reporter protein was confirmed by fluorescence microscopy 24 hours p.i. (data not shown). For quantification and titration of transduction ratio, the cells were infected at MOI's between 50 and 10000 and for practical reasons the transduction efficiency (as % GFP expressing cells) and expression level (as relative units, R.U.) were analysed 48 hours p.i. using a chip-based flow cytometry application [53]. Without further optimisation of the infection and expression conditions, the transduction ratio increased in a dose dependent manner with increased MOI to a maximum of 35 % (Table 2). However, when butyrate was added to the cells to enhance protein expression at a final concentration of 10 mM, the relative amount of GFP expressing-, and thus apparently transduced cells, was significantly increased from about 35 to 82 %. In addition, the maximum relative mean expression level increased more than 4-fold in the SHSY-5Y cells when butyrate was present (Table 2). Butyrate is a histone deacetylase (HDAC) inhibitor, inducing a hyperacetylation of chromatin and enhancement of transcription. The importance of the chromatin state of the baculovirus genome in the transduced cells for transgene expression and the role of HDAC inhibitors has been well described [30,47] and will not be further discussed. Besides the more thorough investigation of SHSY-5Y cells, Chinese Hamster Overy cells (CHO.hIR) stably expressing the human insulin receptor [42], Human Embryonic Kidney cells (HEK293), and rat Fao- and human C3A hepatic-derived cell lines, were screened for the ability to be transduced by BacGFP (Table 3). This screen indicated that the CHO.hIR and the human HEK293 cell lines were easily transduced by BacGFP as previously been reported by others [30]. However, under our conditions butyrate was required to confirm efficient transduction in the CHO.hIR cell line. Whether this was conditional or caused by the heterologous IR was not further investigated. The human hepatic-derived C3A cell line was also easily transduced by BacGFP and similarly to HEK293 and CHO.hIR, butyrate enhanced the expression of GFP and thus the apparent transduction ratio. By contrast, only minor GFP expression was detected in the rat hepatic-derived cell line Fao (Table 3) and was essentially unaffected by the addition of butyrate. Thus rat FAO hepatoma cells appear to be relatively resistant to BacMam viral transduction.

**Table 2:** Transduction of neuroblastoma SHSY-5Y cells with recombinant baculovirus (BacGFP) and influence of butyrate.

	w/o Butyrate		With Butyrate	
	
MOI	Transduction (%)*	Expression level (R.U)	Transduction (%)*	Expression level (R.U)
10000	33 ± n.d	4.8	82 ± n.d	21
5000	35 ± 2	4.0	77 ± 1	14
1000	16 ± 5	3.3	51 ± 3	8.5
500	10 ± 2	2.6	37 ± 7	7.6
100	1.8 ± 1	1.8	16 ± 2	4.6

*Values are given with S.D. for n = 2.

**Table 3:** Transduction of mammalian cell lines with BacMam at different MOI

	Transduced (%)					
	
**Cells**	**MOI 50**		**MOI 100**		**MOI 200**	
	-But	+But	-But	+But	-But	+But
	
CHO.hIR	0.4	50	0.8	75	1.2	91
HEK293	23	97	32	97	52	98
Fao	0.5	0.9	0.5	1.1	0.3	1.8
C3A	12	82	23	89	30	88

Next, the influence of baculovirus infection and recombinant protein expression on the phosphorylation of Akt was investigated. In these studies SHSY-5Y and C3A cells were infected with BacGFP and total and phosphorylated Akt were analysed using an Akt specific ELISA, as previously reported with AdGFP. Baculovirus titres of 1000 and 10000 were chosen to achieve experimentally desired transduction ratios. In contrast to cells infected with adenovirus at MOI 10 and 50, where PKB activation was observed (Table 1), cells transduced with BacMam at MOI 1000 and 10000 showed no apparent increase in PKB phosphorylation. This was true for both SHSY-5Y and C3A cells (Table 1; BacGFP). Taken together, the results from the experiments summarized in Table 1 show that when transduced with adenovirus at MOI 10 and 50, SHSY-5Y and C3A cells respond with an increased phosphorylation of Ser473 Akt (which is not accompanied by an increased expression of Akt) and that this can be avoided by utilizing baculovirus transduction, at MOI's as high as 10000.

### Baculovirus mediated gene transduction and recombinant protein expression do not impair insulin mediated phosphorylation of Akt in SHSY-5Y and C3A cells

**Figure 3:**
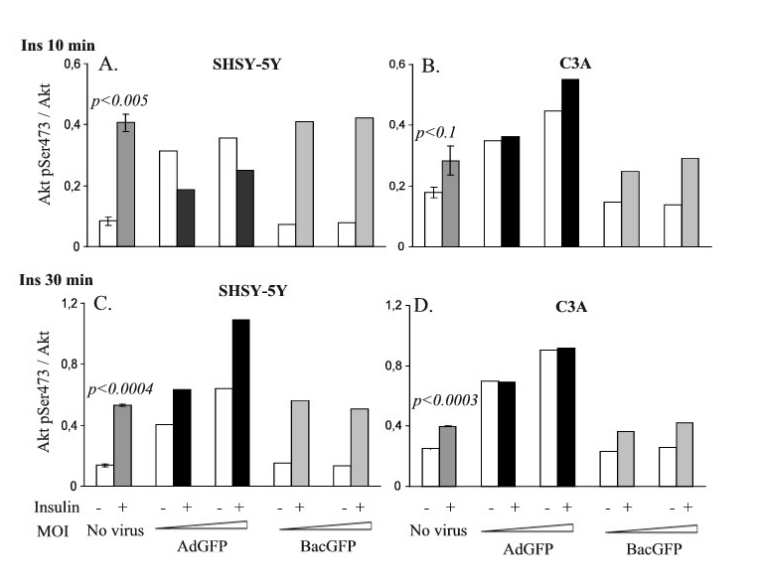
**Insulin stimulation of SHSY-5Y and C3A cells infected with adenovirus or baculovirus vectors**. Cultured SHSY-5Y (A and C) and C3A (B and D) cells were transduced with adenovirus (AdGFP) at MOI10 and 50 or baculovirus (BacGFP) at MOI1000 and 10000, respectively, and the responsiveness to insulin was determined. Non-infected (No virus) and infected cells were treated with 100 nM insulin for 10 (A and B) and 30 minutes (C and D), respectively. Cells were harvested after incubation for total cellular protein, and ELISA was performed with antibodies specific for Akt phosphorylated on serine 473 and total Akt, respectively.

Since infection of SHSY-5Y and C3A cells with baculovirus apparently did not influence the phosphorylation of Akt, the response to insulin following infection was investigated. First the response to insulin of uninfected cells was determined. SHSY-5Y and C3A cells were incubated with 100 nM insulin for 10 and 30 minutes, respectively and the phosphorylation of Akt was determined with phospho-specific ELISA (Figure 3; no virus). The response was most pronounced for SHSY-5Y cells with a transient 4.9-fold increase in Akt phosphorylation 10 minutes after addition of insulin which remained 3.1 – fold elevated after 30 minutes (Figure 3A and 3C, no virus). This data was again verified for SHSY-5Y cells with western blot using specific Ser^473^-phosho-Akt antibodies (Figure 2, lane 1 and 2). The C3A cells also responded to insulin albeit to a lower degree reaching a sustainable 1.6 – fold increase in Akt phosphorylation after 10 and 30 minutes incubation, respectively (Figure 3B and 3D, no virus). Next, the effect of insulin stimulation of cells infected with adenovirus and baculovirus, respectively, were investigated. As previously found for SHSY-5Y cells infected with adenovirus (Table 1; Figure 2, lane 1 and 3), infection with AdGFP at MOI10 and 50 in the absence of insulin gave a dose dependent increase in Akt phosphorylation (Figure 3A and 3C). In addition, when the cells were incubated in the presence of 100 nM insulin for 10 minutes no additional phosphorylation of Akt was obtained (Figure 3A; Figure 2, lane 3 and 4). After 30 minutes of incubation with 100 nM insulin, Akt was further activated 1.6 and 1.7-fold over the activation already induced by adenovirus infection (Figure 3C). By contrast, SHSY-5Y cells infected with baculovirus showed no sign of basal Akt phosphorylation after 10 or 30 minutes (Figure 3A and 3C). Moreover, upon insulin stimulation of baculovirus infected cells, the response was analogous to that for non infected cells incubated with insulin. At 10 minutes incubation, a 5.7 and 5.4-fold increased phosphorylation was obtained at MOI1000 and 10000 (Figure 3A), and at 30 minutes incubation a 3.6 and 3.8-fold increase in Akt phosphorylation was obtained at MOI1000 and 10000, respectively (Figure 3C). Qualitatively similar data were obtained for C3A cells (Figure 3B and 3D). Thus adenovirus infection increased basal Akt phosphorylation and insulin was not able to increase this further. By contrast, BacMam-transduced cells displayed no elevated basal Akt phosphorylation and insulin was able to induce a 1.6 to 2.1-fold activation of the enzyme, as for control cells. The apparently smaller Akt response in C3A cells compared to SHSY-5Y cells may be related to the higher basal level of Akt phosphorylation in unstimulated cells and is a feature of other hepatoma cells lines in culture including rat FAO and human HepG2 cells (data not shown). Interestingly, when we sought to examine the effects of virus infection on ERK1/2 activity in hepatoma cells, we found elevated basal ERK1/2 phosphorylation such that further increases could not be observed (data not shown). Thus our data show that cells transduced with baculovirus remain responsive to insulin stimulation as measured by phosphorylation of Akt.

## Discussion

In our earlier work, we reported that common lipid transfection reagents are able to induce a state of cellular insulin resistance which hampers significantly subsequent experiments designed to examine insulin signal transduction [42]. We hypothesised that the activation of the insulin receptor which is causal to this state of insulin unresponsiveness is due to lipid phase separations in the plasma membrane. In the current work, we have confirmed that the use of adenoviral transduction vectors in place of lipid transfection reagents leads to activation of components of the insulin signalling PI3K pathway, with subsequent induction of a state of insulin unresponsiveness. In this case, the mechanistic basis of the effects of adenovirus transduction are better established, with the potential involvement of integrin proteins at the cell surface leading to PI3K activation [12]. Alternatively, it was recently shown by O'Shea and co-workers that adenovirus encodes two proteins that activate the mammalian target of rapamycin (mTOR) and that one of these (E4-ORF1) mimics growth factor signalling by activating PI3K [55].

In our search for transduction agents of recombinant DNA that do not affect the cellular insulin signalling apparatus, we examined the effects of the BacMam baculovirus vector encoding the model protein GFP. Although known for a long time that baculoviruses can efficiently be taken up by mammalian cells [48], the mode and kinetics of baculovirus infection of which are poorly understood. Recently, evidence for clathrin-mediated endocytosis has emerged as a major entry route in mammalian cells [49,50]. Our data show that this vector is effective as a transduction agent of mammalian cells in culture and that it has no apparent effect on components of the insulin signalling cascade leading to the activation of PKB. As such, it does not induce a state of insulin unresponsiveness and may be a promising tool for investigations of recombinantly-expressed proteins on insulin signal transduction. Possible reasons for why BacMam transduction avoids cellular insulin resistance would include lack of interaction with surface integrin proteins and the fact that the heterologous virus does not hijack cellular protein translational machinery to the same degree as adenovirus. It is however possible that that transient effects on PKB activation and other insulin signalling events upon baculovirus transduction may still be possible and may occur at time points that we have not mapped. However, the major advantage of the baculovirus system is that cells remain responsive to insulin stimulation showing clear activation of PKB after transduction with the virus, which is not possible with adenovirus vectors.

Interestingly, the effectiveness of BacMam as a transduction agent is greatly enhanced in the presence of the HDAC inhibitor butyrate, which presumably leads to enhanced chromatin modification and facilitates DNA transcription. Differential repression of baculovirus mediated transgene expression in different cell types is well known and the use of HDAC inhibitors such as butyrate and trichostatin A are widely use to increase gene expression [30]. Considering that HDAC2 is a negative regulator of insulin signalling via interactions with insulin receptor substrate proteins and that global inhibition of HDAC activity enhances insulin signalling [51], it is unlikely that BacMam transduction in the presence of HDAC inhibitors will be appropriate for subsequent insulin signalling studies. However, considering the relatively benign effects of BacMam exposure on cellular well-being, it may be possible to increase MOIs further should higher expression levels of the delivered gene of interest in the studied cell line be required. In addition, individual cell lines demonstrate different susceptibilities to transduction by baculovirus (Table 3) and therefore transduction conditions such as virus dosage, temperature, viral exposure time, and transduction media should be optimized for individual cell lines in order to increase the transduction efficiency [56], Finally, baculovirus vectors utilizing enhancer elements such as hr1 [57] and episomal OriP/EBNA-1 elements [58] in combination with mammalian promoters have been shown to increase and prolong transgene expression in mammalian cells.

## Conclusion

The BacMam baculovirus vector is a promising reagent for the transduction of mammalian cells in culture allowing subsequent insulin signalling studies. The virus does not induce a state of insulin unresponsiveness as do adenovirus vectors and lipid transfection reagents. As such, BacMam is an agent of choice to examine the effects of recombinantly expressed proteins on insulin signal transduction.

## Methods

### Materials

All cell culture reagents were from Invitrogen. Hybond-C nitrocellulose membrane and enhanced chemiluminescence (ECL) were from Amersham Life Science. BCA protein analysis kit was from Pierce. Anti-phosphoserine and protein kinase B/Akt anti-bodies and Path Scan™ total Akt and Ser^473 ^Phospho-Akt ELISA kits were from Cell Signalling Technology.

### Generation of recombinant adeno- and baculovirus

The pADTrack-CMV and pADEasy-1 plasmids, and *E. coli *strain BJ5183 used to construct recombinant adenovirus were obtained under license agreement from Dr Bert Vogelstein, John Hopkins Oncology center, Howard Hughes Medical Institute (Baltimore, USA) [54]. To generate the vector pMB1039, homologous recombination of approximately 1 μg *PmeI *digested shuttle vector (pADTrack-CMV) and 0.1 μg supercoiled ad5 backbone vector (pAdEasy-1) was performed with 40 μl electrocompetent BJ5183 (endA sbcBC recBC galK met thi-1 bioT hsdR (Str^r^)). The BJ5183 cells were grown to OD_550 _0.8, then collected and washed twice with ice-cold 10% glycerol. Electroporation was performed in 2.0 mm cuvettes at 2500 V, 200 ohms, and 25 μF in a Bio-Rad Gene Pulser electroporator. The mixture was then plated onto agar-kanamycin media. Only the smallest colonies were selected for screening by restriction analysis prepared from mini-preps. Linearized pMB1039 was subsequently transfected into adherent HEK293A cells in MEM containing Earle's salts and glutamine and supplemented with 10 % FCS. Virus was plaque purified and subsequently propagated in HEK293A cells in E-MEM supplemented with 2 % FCS. Amplified virus was purified using CsCl density gradient ultracentrifugation essentially as described previously [52]. Isolated virus was dialysed twice in D-PBS/10 % glycerol and finally diluted in storage buffer containing 100 mM Tris-HCl (pH 8), 100 mM NaCl, 0.1 % BSA, and 50 % glycerol. Titer determination was performed by end point dilution on HEK293 cells and adenoviral stocks used for the experiments were typically 10^10 ^pfu/mL.

The BacMam vector was designed essentially as described by Condreay and coworkers [30]. To construct this vector, a fragment containing the CMV-IE promoter/enhancer, multiple cloning site and polyadenylation signal was isolated from the pcDNA3.1+ vector (Invitrogen) by PCR using primers 5'-CGCGTCCGGAGGGCCAGATATACGCGTTGACATTG-3' (JN143) and 5'-CCCCCTAGAGCCCCAGCTGGTTC-3'. In parallel, a fragment containing the SV40 promoter-neomycin phosphotransferase II expression cassette was isolated by PCR from the pcDNA3.1+ vector using primers 5'-GAACCAGCTGGGGCTCTAGGGGGCTGTGGAATGTGTGTCAGTTAGGGTGTG-3' and 5'-CCCCAAGCTTCTCAGAAGAACTCGTCAAGAAGGCG-3' (JN146). The fragments were assembled by overlapping PCR using the JN143 and JN146 primers which contains a *Bsp*EI and a *Hin*dIII restriction site (underlined), respectively, and inserted between *Bsp*EI and *Hin*dIII in pFastBac1 (Invitrogen). The constructed Bacmam vector was named pBV973. A gene fragment encoding GFP was subsequently inserted between *Kpn*I and *Bam*HI in pBV973

Recombinant BacGFP virus was generated using the Bac-to-Bac system (Invitrogen) essentially as described [30]. Briefly, propagation of virus was performed in Sf-9 insect cells maintained as suspension cultures in SF900-II SFM (Invitrogen) at 27°C. For amplification to second generation virus used in the experiments, the Sf9 cells were infected at MOI 0.1 at a cell density of 2 × 10^6 ^cells/mL. 5 days after infection, the culture medium containing amplified virus was harvested and titres were determined using the BacPAK baculovirus rapid titer kit (BD Biosciences, Clontech, Clontechniques XII(3): 8–9, 1997). In this titering method, a primary monoclonal antibody raised to an AcMNPV envelope glycoprotein (gp64) was used to label infected cells in replicate samples. A secondary HRP-conjugated antibody was used to stain the infected cells so the number of infected foci could be determined under light microscope. Viral titers between 10^8^–10^9 ^pfu/mL were routinely obtained. Stocks of virus for experiments in mammalian cells were concentrated by centrifugation at 35000 × g for 60 minutes and pelleted virus was resuspended in Dulbecco's PBS supplemented with FBS (1 vol-%) to a final concentration of 10^10 ^pfu/mL.

### Cell culture and treatment with adeno- and baculovirus

C3A cells were cultured in MEM supplemented with 10% heat inactivated FCS, 1% non-essential amino acids and 1% Na-pyruvate. SHSY-5Y cells were cultured in EMEM: Ham's F12 (1:1) supplemented with 15% heat inactivated FCS and 1% non-essential amino acids. Both cells lines were kept at 37°C in a humidified 5% CO_2 _atmosphere. One day prior to experiment, cells were seeded out into 6 well plates (0.25 × 10^6 ^cells/well) and cultured over night. Adeno- and Baculo-virus stocks were diluted accordingly and added to the wells. Medium was replaced with fresh serum-free medium after 24 h. Where indicated in Table 2 and 3 for the expression studies, 10 mM butyrate was added to the incubation medium. After additional 24 hour incubation under serum-free conditions, medium was replaced with fresh serum-free medium with or without (control) 100 nM insulin for 10 and 30 minutes, respectively. The plates were then placed on ice, and the cells were washed with ice cold PBS prior to harvest, using lysis buffer included in the ELISA kit (20 mM Tris-HCl, pH 7.5, 150 mM NaCl, 1 mM Na_2_EDTA, 1 mM EGTA, 1% Triton, 2.5 mM Na-pyrophosphate, 1 mM β-glycerophosphate, 1 mM orthovanadate, 1 μg/ml leupeptin). The lysis buffer was supplemented with Complete™ protease inhibitor cocktail (Roche). As indicated in some experiments, cells were stimulated with 100 nM human recombinant insulin (Sigma) for 10 or 30 min prior to harvest. Lysates were then stored at -70°C prior to analysis. Total protein content was determined using Pierce BCA protein analysis kit.

### Sampling and analytical

For western blot analyses, samples were matched for protein concentration and heated at 70°C for 10 minutes with reducing sample buffer. Samples were resolved on 4–12 % gradient gels and transferred to nitrocellulose membranes. Membranes were probed for the presence of total and phosphorylated Akt using antibodies obtained from Cell Signaling Technology, and developed using enhanced chemoluminesence. The signal was then quantified by densitometric analysis. Alternatively, total and Ser473 phosphorylated Akt was determined with a commercially available ELISA system for PKB/Akt from Cell signalling Technology.

### Flow cytometry analysis of green fluorescent protein expression

The flow cytometry data presented in Figure 1 and Table 3 was processed using an EPICS XL-MCL flow cytometer (Beckman-Coulter) essentially as described previously [42]. For the experiments in Table 2, protein expression was measured with an Agilent 2100 Bioanalyzer using the chip-based flow cytometry application [53]. Briefly, cultures were washed with PBS, harvested with cell dissociation buffer (500 μL for 1 min) and re-suspended in 500 μL OptiMem (Invitrogen) at room temperature. Staining for viable cells was performed by adding carboxynaphtofluorescein (CBNF, Molecular Probes, Inc., Leiden, NL) at a final concentration of 0.5 μM. After 15 minutes, cells were centrifuged at 500 × g for 5 minutes, followed by an additional washing and centrifuge step and finally the pellet was re-suspended in 400 μL Cell buffer (Agilent Technologies). 10 μL cell samples were applied to the chip and analyzed utilizing the GFP cytometry mode. Data was obtained by gating for viable cells and adjusting for background fluorescence. Values were determined for viable GFP expressing cells.

## Authors' contributions

MA carried out infections with baculovirus and adenovirus in treated cells, performed phosphorylation and flow cytometric analyses, and participated in the design of experiments. MW and CSt carried out initial characterization of adenovirus infections and comparative analyses of protein phosphorylation. JN, CS, and KB were involved in the design and molecular cloning of constructs. MS performed insect cell cultures and baculovirus propagation and preparation. SRJ participated in the design of PKB experiments, the design of the study and preparation of the manuscript. MD conceived of the study, designed experiments and played a coordinative role in experiments and preparation of a draft manuscript. All authors read and approved the final manuscript.
